# Necrotizing Enterocolitis Complicated by Hepatic Abscesses in a Neonate: Diagnostic Utility of an Abdominal Ultrasound

**DOI:** 10.7759/cureus.81587

**Published:** 2025-04-01

**Authors:** Kenichi Takahishi, Takahiro Kido, Yuki Okada, Takuma Deguchi, Yoshihiro Nozaki, Yayoi Miyazono, Hidetoshi Takada

**Affiliations:** 1 Department of Pediatrics, University of Tsukuba Hospital, Tsukuba, JPN; 2 Department of Child Health, Institute of Medicine, University of Tsukuba, Tsukuba, JPN

**Keywords:** infant, liver abscess, necrotizing enterocolitis, screening, ultrasonography

## Abstract

We report a case of multiple liver abscesses after necrotizing enterocolitis (NEC), which were successfully detected by ultrasound screening and treated successfully. This baby was not premature but had truncus arteriosus and was at risk of NEC. NEC developed on day 7, and abdominal drainage, bowel resection, and enterostomy were performed on day 12. The postoperative clinical course and laboratory data were uneventful. However, abdominal ultrasound screening, which was performed to check for any postoperative complications, revealed multiple liver abscesses on postoperative day 14. Contrast-enhanced computed tomography was performed at the same time, but only one of them was detected. Broad-spectrum antibiotics were administrated intravenously for eight weeks in total. Follow-up ultrasounds showed only scars by day 79. The prognosis for neonates with multiple liver abscesses after NEC is poor, but they could be detected by ultrasound and successfully treated while asymptomatic in this case. High spatial resolution for small lesions and noninvasiveness to patients make ultrasound suitable for screening neonatal liver abscesses. Neonatal NEC can cause fatal complications. The development of workup protocols, including ultrasound screening, may contribute to improved prognosis.

## Introduction

Neonatal liver abscess related to necrotizing enterocolitis (NEC) is a rare but potentially life-threatening condition [1,2]. Symptoms may not be present in the early stages, and when they do appear, they are often non-specific, such as sepsis, elevated liver enzymes, and hepatomegaly, making the diagnosis of NEC-related neonatal liver abscess difficult [3]. However, strategies for detecting liver abscesses after neonatal NEC have not been discussed. In infants, ultrasonography is a sensitive diagnostic test for detecting liver abscesses and is beneficial because it can be performed repeatedly at the bedside without radiation exposure [4]. In our institution, ultrasound screening is performed to check for complications after abdominal surgery. Here, we describe a neonatal case of multiple asymptomatic liver abscesses secondary to surgery for NEC detected by ultrasound screening.

## Case presentation

A male infant was born via vaginal delivery at 38 weeks and six days of gestation with a birth weight of 2,855 g and Apgar scores of 8 at 1 min and 9 at 5 min. The mother had no previous medical history, and the gestational course was unremarkable. Shortly after birth, the patient was transferred to our hospital owing to hypoxia and was diagnosed with truncus arteriosus. An abdominal ultrasound examination at hospitalization did not reveal any other visceral malformations. Despite limited oxygen and noninvasive positive pressure ventilation, high pulmonary perfusion progressed to heart failure. Hypoxic gas therapy using nitrogen was started on day 5, and catecholamine was also administered; however, NEC developed on day 7. No gastrointestinal perforation occurred at this point, and pulmonary artery banding was performed the same day to avoid further bowel ischemia. However, owing to bowel perforation, the patient required abdominal drainage, bowel resection, and enterostomy on day 12, and intravenous meropenem administration was initiated.

**Figure 1:**
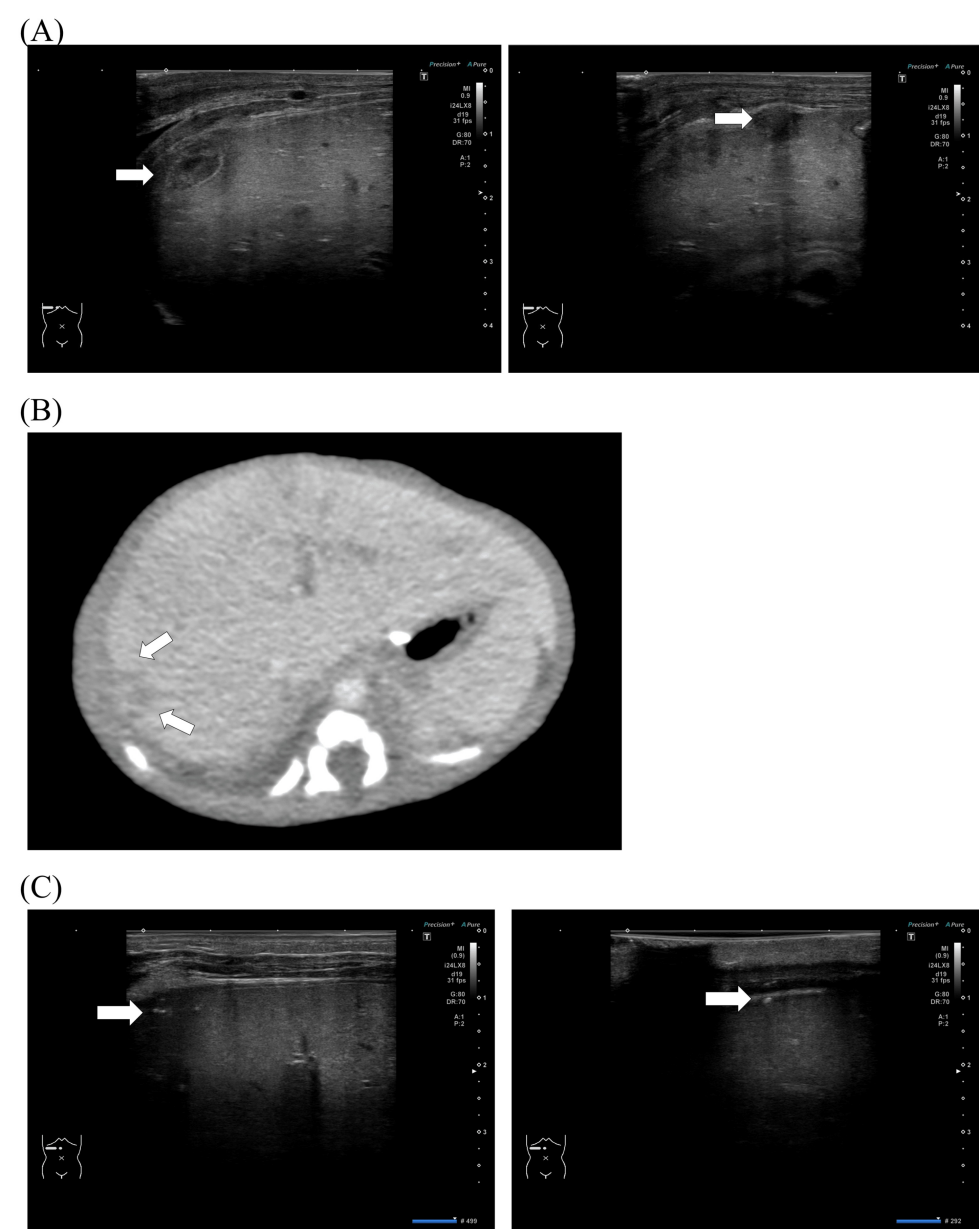
Ultrasound and computed tomography images of liver abscesses during the course of the disease. (A) Abdominal ultrasound performed on day 23 of life. Liver abscesses were detected in the liver parenchyma (arrow), with one in segment S7 (left) and the other in S8 (right) of the hepatic zone, measuring 20 mm and 5 mm in diameter, respectively. The irregular, low-echogenic heterogeneous lesions with irregular margins were consistent with abscesses. (B) Abdominal contrast-enhanced computed tomography (CT) performed on day 26 of life. CT indicated only a single low-density 15 mm nodule (arrow), while four lesions were observed on the abdominal ultrasound performed on the same day. (C) Abdominal ultrasound performed on day 79 of life. Liver abscesses in segments S7 (left) and S8 (right) had shrunk and became organized (arrow).

The post-operative course had seemed to be uneventful until the ultrasound was performed on the 14th post-operative day. The laboratory inflammation marker peaked on the second postoperative day and then showed a gradual downward trend. Clostridium species were detected in the blood and ascites, and antibiotics were planned to be de-escalated to ampicillin. Abdominal ultrasound screening was performed at 3, 7, and 14 days postoperatively to check for signs of postoperative complications, such as stricture, ileus, and anastomotic leaks. No fluid retention was present in the abdominal cavity, and the initially poor peristalsis of the small bowel recovered over time. Enteral feeding was resumed on postoperative day 9. On day 23 of life, 14 days postoperatively, an ultrasound examination revealed new hypoechoic lesions in the liver parenchyma (Figure 1A). One lesion was located in segment S7 and the other in segment S8 of the hepatic zone, measuring 20 mm and 5 mm in diameter, respectively. No internal blood flow was detected. A diagnosis of liver abscesses was made based on the identification of irregular, low-echogenic heterogeneous lesions with irregular margins. Contrast-enhanced computed tomography (CT) performed on day 26 revealed only a single nodule at S7 (Figure 1B); however, an ultrasound performed on the same day identified four abscess lesions in segments S8 (6 mm), S7 (15 mm), S6 (4 mm), and S5 (5 mm). As the patient had multiple abscesses, it was decided not to perform drainage. We decided to quit de-escalation to ampicillin for antimicrobials and switch to meropenem for two weeks and then to piperacillin and tazobactam for a minimum of eight weeks in total. Follow-up ultrasounds performed every few days confirmed the shrinkage and organization of the lesions by day 79 (Figure 1C).

The patient was tested for immunodeficiency disorders. Serum IgG levels, neutrophil bacteriocidal capacity test, and genetic screening for primary immunodeficiencies revealed no abnormalities. In addition, chromosome G-banding, fluorescence in situ hybridization (FISH) test for 22q11.2 deletion syndrome, and assessments of neutrophil phagocytosis and migration capacity were normal. No subsequent flare-ups of liver abscesses occurred after the antimicrobials were terminated. However, the patient died at seven months during the treatment course for cardiac defect.

## Discussion

In the present case, the liver abscesses were detected by abdominal ultrasound during the postoperative course of NEC despite the absence of specific symptoms. This case demonstrates the significant impact on the management strategy of timely and detailed ultrasound assessment.

To date, no protocol has been established for the early detection of complications after NEC. The overall incidence of acute complications after neonatal NEC surgery is known to be high, with stenosis occurring in 30% of cases, followed by recurrent NEC and adhesive ileus [5]. In recent years, the efficacy of bedside ultrasound has received increasing attention and is becoming established as a modality used by physicians in NICUs. All these complications are considered amenable to clinical judgment with ultrasound [6]. Ultrasound may be the most appropriate device for screening for post-NEC complications. Although the frequency of liver abscess after NEC is low, detailed below, the mortality rate is as high as 15-50% [7], and patients should be screened for it, as early detection may improve prognosis [8].

The disease course of neonatal liver abscesses seems to depend largely on the number of lesions. To date, only eight cases of liver abscess after NEC have been reported in the English literature, including this case. The characteristics of these cases are summarized and presented in Table 1. There have been no reported cases of asymptomatic liver abscesses detected after NEC. Three out of five cases with a single liver abscess survived [2,3,9], whereas multiple liver abscesses had a worse prognosis, and all but the present case died [1]. The two cases of multiple liver abscesses reported to date were affected by immaturity, making it difficult to compare the course of the disease [1]. However, given that multiple neonatal liver abscesses are generally associated with sepsis and have a poor prognosis [10], detection and intensified treatment in this case, while the patient was asymptomatic, might have contributed to the outcome [10].

**Table 1:** Reported cases of neonatal liver abscess related to necrotizing enterocolitis (NEC). *Day indicates the respective day of age at the time NEC and liver abscess were diagnosed. **Pathogens identified in cultures of blood, ascites fluid, or abscess. MRSA, methicillin resistant staphylococcus aureus; US, ultrasound; CT, computed tomography; n.d., no data available.

					NEC		Liver abscess							
Single/multiple abscess	Case No.	Gestation (weeks)	Birth weight (g)	Sex	Day*	Treatment	Day*	Sepsis	Imaging modality	Other symptom	Isolated agent**	Treatment	Outcome	Reference
Single	1	-	3400	Male	6	Surgery	14	-	US	Tense abdomen and blood test abnormality	Escherichia coli	Drained	Survive	Lim & Koh (1994) [9]
	2	29	1470	Female	15	Conservative	18	-	US	Abdominal distension	Staphylococcus hominis	Conservative	Survive	Semerci et al. (2016) [2]
	3	25	865		11	Conservative	15	-	US/CT	Frequent apnea, abdominal distension	MRSA	Conservative	Survive	Semerci et al. (2016) [2]
	4	34	1373	Female	-	Surgery	n.d.	n.d.	No (autopsy)	n.d.	Klebsiella sp.	n.d.	Died	Moss & Pysher (1981) [3]
	5	32	1200	Male	n.d.	Conservative	n.d.	n.d.	No (autopsy)	n.d.	Candida sp.	n.d.	Died	Moss & Pysher (1981) [3]
Multiple	6	27	481	Female	n.d.	n.d.	60	+	US	Abdominal distension	MRSA	Conservative	Died	Tan et al. (2015) [1]
	7	24	660	Male	n.d.	n.d.	11	+	US	Abdominal distension	Klebsiella sp. and Candida albicans	Conservative	Died	Tan et al. (2015) [1]
	8 (our case)	38	2855	Male	7	Surgery	15	-	US/CT	Not symptomatic	Clostridium sp.	Conservative	Survive	Our case

CT and MRI were insufficient in this respect. In this case, CT scans could only detect lesions measuring more than 1 cm in diameter. Although CT was performed at a thickness of 1 mm, which theoretically allows for the detection of lesions that are a few millimeters in size, the temporal resolution may not have been sufficient for a neonate with a high respiratory rate. As shown in Table 1, previous case reports have also used ultrasound findings to detect liver abscesses.

A limitation of this case is the lack of microbiological confirmation from abscess aspiration. However, aspiration is not always clinically necessary, especially in neonates, where invasive procedures should be minimized.

Although no post-NEC screening protocol has been established, follow-up with an ultrasound every few days until one to two weeks after early complications are expected to occur may be advisable.

## Conclusions

A case of multiple liver abscesses after NEC, which were successfully detected by ultrasound screening while the patient was asymptomatic, is reported. This case illustrates the effectiveness of ultrasound in detecting post-NEC complications in neonates by avoiding the highest risk conditions. The development of testing protocols, including ultrasound screening of neonates after NEC, may contribute to prognosis.
